# Adequacy of the Type of Venous Catheter to the Drug Type and Duration of Treatment: A Cross-Sectional Study

**DOI:** 10.3390/nursrep16020076

**Published:** 2026-02-21

**Authors:** Esther Moreno-Rubio, Carlos Pérez-López, João Carmezim, David Blancas-Altabella, Antonella F. Simonetti, Silvia Serda Sanchez, Alejandro Rodríguez-Molinero

**Affiliations:** 1 Infectious Diseases Department, Consorci Sanitari de l’Alt Penedès i Garraf, Vilafranca del Penedès, 08720 Barcelona, Spain; emoreno@csapg.cat (E.M.-R.); dblancas@csapg.cat (D.B.-A.); 2 Research Department, Consorci Sanitari de l’Alt Penedès i Garraf, Vilafranca del Penedès, 08720 Barcelona, Spain; cperezl@csapg.cat; 3 Unidad de Bioestadística, Institut de Investigació Biomédica de Bellvitge (IDIBELL), L’Hospitalet de Llobregat, 08907 Barcelona, Spain; joaocarmezimcorreia@gmail.com; 4 Infectious Diseases Department, Hospital de la Santa Creu i Sant Pau, 08025 Barcelona, Spain; antonella.f.simonetti@gmail.com; 5 Pharmacy Department, Consorci Sanitari de l’Alt Penedès i Garraf, Vilafranca del Penedès, 08870 Barcelona, Spain; sserda@csapg.cat

**Keywords:** intravenous therapy, central venous catheter, peripheral venous catheter, adequacy

## Abstract

**Background**: Venous catheters are standard devices in clinical practice. However, their use is not exempt from possible errors and complications. In addition, using them effectively is key to avoiding complications such as infection or phlebitis. **Objectives**: To determine the frequency of appropriate venous catheters chosen based on the drug and treatment duration in hospitalized patients in a region with 154,000 inhabitants. **Methods**: A cross-sectional design was carried out between 14 and 28 February 2020, in patients with a peripheral or central intravenous catheter admitted to the acute care unit. Variables collected were related to the catheters, patients, and nurses. **Results**: One hundred and eighty-eight patients were included, with 319 catheters inserted by 68 nurses. Seventeen patients (8.8%) were ruled out due to the lack of data on the medication administered. Finally, data from 171 patients were included in the final analysis, with 297 catheters inserted. Of them, 246 catheters (82.8%) were inadequate. **Discussion**: In this point-prevalence study, catheter inadequacy affected more than four out of five catheters and was mainly linked to the use of peripheral catheters for high-risk IV medications and/or for treatments extending 7 days or more. **Conclusion:** The selection of venous catheters in acute care units is not usually adequate since many peripheral catheters are placed in patients who require intravenous medication during a prolonged period or who are receiving risk medication.

## 1. Introduction

Intravenous therapy is one of the essential clinical practices used in hospitalized patient care when the medication cannot be administered orally [1,2,3]. A central or a peripheral line can be used for diagnostic purposes, such as to administer radiological contrasts, or therapeutic purposes, as in the case of intravenous fluids or drugs. Its main difference lies in the venous catheter insertion route and the location of the distal end. The peripheral venous catheter (PVC) must be placed in the limbs, and in the case of the central venous catheter (CVC), the end of the catheter should be positioned at or near the cavoatrial junction or within the lower third of the superior vena cava [4]. 

Venous catheters are very common in clinical practice. It is estimated that approximately 33–67% of hospitalized patients require at least one PVC [5]. The Study of the Prevalence of Nosocomial Infections in Spain (EPINE) [6] of 2023 revealed that 76.2% of hospitalized patients had a PVC and 12.5% had a CVC at the moment of data collection. According to data from the Nosocomial Infection Surveillance Program in Catalan hospitals (VINCAT) for the same year, 78.6% of hospitalized patients had a vascular catheter (77.7% peripheral and 5.9% central) [7].

The use of intravenous catheters is not exempt from possible errors and complications [8,9]. Their use for prolonged periods entails a high risk of local and systemic infections [10,11]. The insertion of a foreign body causes an inflammatory reaction that can render the vein useless, forcing nurses to find another vein suitable for catheterization [12]. Peripheral venous access may be exhausted in patients who frequently require this form of treatment, leading to a risk for patient care [13,14]. Therefore, preserving the patient’s venous field is essential since it is unrecoverable. The correct choice of path, sterility at the time of insertion, checking that the catheter stay does not exceed the recommended time, and extreme care when handling and maintaining the catheter are the main factors that help reduce infection rates [15].

The most frequent complications occur during the catheter insertion or during its handling and maintenance and are usually occlusion, mechanical, chemical, or bacterial phlebitis, extravasation, and infection [11,16]. Phlebitis, an inflammation of the venous wall, occurs in 13–56% of hospitalized patients [17]. The correct choice of the catheter is key to avoiding complications that can result from prolonged use of catheters, such as infection or phlebitis, or even more severe adverse events, such as sepsis [18,19]. Despite the standardized algorithms for catheter selection, the level of compliance with recommendations and the training of nurses has been scarcely investigated [20]. The study’s main objective was to determine the frequency of adequate venous catheters being chosen based on the drug and treatment duration in inpatients of a second-level hospital with a reference population of 154,000 inhabitants and comprising 150 acute care beds and 16 intensive care unit beds.

## 2. Materials and Methods

### 2.1. Study Design

The study was carried out at Hospital Sant Camil (Sant Pere de Ribes, Spain) and had a cross-sectional design. Four inpatient wards (acute care units) participated in the study. Data were collected between 14 and 28 February 2020. The data were pseudonymized for analysis.

Primary Objective: To determine the type of venous catheter adequacy based on the prescribed drug and the treatment duration.

Secondary Objectives: (1) To identify the factors that determined the adequacy of the choice of an intravenous catheter; (2) to learn about the autonomy of the nurse professionals to choose the type of catheter.

### 2.2. Subjects

Study participants were the inpatients and the assigned nurses who cared for them during the study.

Patients met the following selection criteria:

Inclusion criteria: (1) Patients with a peripheral or central intravenous catheter; (2) patients admitted to the acute care units; (3) patients admitted from 14 to 28 February 2020, at Hospital Sant Camil (Sant Pere de Ribes, Spain).

Exclusion criteria: patients admitted to pediatric hospitalization units, ICU, or the Emergency Service.

All nurses included in the study were registered nurses who had completed the official undergraduate nursing degree program in Spain, equivalent to a Bachelor of Science in Nursing. As part of the hospital’s standard onboarding process, nurses receive theoretical and practical training on venous catheter insertion, maintenance, and removal in accordance with institutional protocols. This training includes a supervised practical period of approximately one week, during which nurses perform catheter-related procedures under supervision, managing a minimum of approximately 40 venous catheters before performing these procedures independently. Supervised training and return demonstrations were conducted by nurses specialized in infection prevention and control.

### 2.3. Variables Analyzed

- Catheter-related variables: Type, including short peripheral catheter (SPC), peripherally inserted central catheter (PICC) or centrally inserted central catheter (CICC), duration of catheterization (catheter dwell time was calculated using recorded insertion and removal dates), type of perfusion administered (pH and osmolarity), the number of catheters inserted and incident complications related to the catheter during the study period: loss of puncture site, extravasation, accidental removal/dislodgement, phlebitis, obstruction, non-patency, or bacteremia.
- Descriptive variables of the patient: Age, sex, medical service that treated the patient (internal medicine, general surgery, traumatology, neurology, cardiology, pulmonology, urology, and geriatrics), and hospitalization days.
- Descriptive variables of the nurse: Age, sex, years of professional practice, autonomy regarding the choice of the catheter, participation in a training program on venous catheters. Participation in a training program referred specifically to the *Flebitis Zero* program, a patient-safety initiative focused on improving venous catheter management and reducing catheter-related complications through standardized training and evidence-based recommendations [21]. Autonomy in catheter selection was assessed using the following self-administered question: “Do you have autonomy to choose the type of catheter to be inserted?” with four response options: “Yes, always”; “Almost always (usually)”; “Almost never (occasionally)”; and “Never.”

### 2.4. Study Procedures and Data Management

Investigators obtained the patient information from the data included in the medical records available at the hospital. All study variables are routinely recorded in predefined, structured fields within the Hospital Information System (HIS); therefore, data extraction was performed automatically from these specific fields using a standardized query. The nurses’ data were obtained from a self-administered questionnaire that included their autonomy to choose the catheter.

The HIS includes a specific nursing documentation module for venous catheters. After each catheter insertion, nurses record the type of catheter, date and time of insertion, the hospital unit where the patient is admitted, the use of complementary devices (e.g., extension lines, three-way stopcocks, or others), and the type of infusion (bolus, continuous infusion, parenteral nutrition, or chemotherapy). During catheter maintenance, the insertion site is inspected once per shift and the result of the inspection is recorded. At catheter removal, nurses document the date and time of removal and the reason for removal, as described in this study.

Catheter adequacy was defined according to previously published recommendations for intravenous therapy selection. Specifically, peripheral venous catheters were considered appropriate when used for short-term intravenous treatments (≤5 days) and for medications with osmolarity ≤ 600 mOsm/L and pH between 4 and 9. For treatments exceeding 5 days or involving medications with extreme pH (<4 or >9) or high osmolarity (>600 mOsm/L), the use of a central venous access device (peripherally inserted central catheter or centrally inserted central catheter) was considered appropriate. These criteria were based on the algorithm proposed by Manrique-Rodríguez et al. (2021) [20].

Insertion of PICC and CICC was performed under ultrasound guidance according to institutional protocols. Short peripheral catheters were inserted following standard clinical practice and were not routinely verified using imaging techniques prior to infusion.

Using the Hospital Information System (HIS), a list of all prescribed intravenous treatments was prepared, by day, type of catheter, and actual duration. All intravenous drugs that were prescribed and administered to the included patients during the study period were considered for analysis, without any prior selection. The route of administration (peripheral or central intravenous) was identified from the prescription and administration records in the HIS. In addition, the medication administered was reviewed to establish its pH and osmolarity and assess whether the catheter was appropriate. The database was purged once all the information was obtained, eliminating extreme values and erroneous data.

Access to medical records was restricted to authorized members of the research team, all of whom were authorized hospital staff. Data extraction was performed after approval by the institutional ethics committee and in compliance with data protection regulations. All records were pseudonymized prior to analysis, and no directly identifiable patient information was accessed or stored.

### 2.5. Sample Size

Sample size was calculated to estimate the proportion of adequate catheter selection. Assuming an expected adequacy prevalence of 90% (*p* = 0.90), a two-sided 95% confidence level (Z = 1.96), and an absolute precision of 5% (d = 0.05), the minimum required sample size to estimate a single proportion was 139 catheter insertions.

### 2.6. Statistical Analysis

The normal distribution of each study variable was analyzed. Descriptive statistical analyses were performed on the characteristics of the patients and nurses, describing the sample using quantitative variables, means, standard deviations and medians, interquartile ranges, categorical variables, frequencies, and percentages.

The proportion of adequately and inadequately selected catheters was calculated with 95% confidence intervals. Associations between catheter adequacy and catheter-, patient-, and nurse-related variables were first explored using bivariate analyses (χ^2^ test or Fisher’s exact test for categorical variables, and Student’s t test or Mann–Whitney U test for continuous variables, as appropriate).

To identify factors independently associated with catheter adequacy, mixed-effects logistic regression models were fitted, considering catheter adequacy as the dependent variable and including patient- and nurse-related variables as fixed effects, with medical service included as a random effect to account for clustering.

To further assess the nurse effect, we calculated for each nurse the proportion of correctly selected catheters out of the total catheters inserted by that nurse. A Poisson regression model was fitted with the number of adequately selected catheters as the response variable, including the total number of catheters inserted by each nurse as an exposure variable (log-transformed), and nurse-related variables as covariates.

## 3. Results

### 3.1. Patients

**Figure 1:**
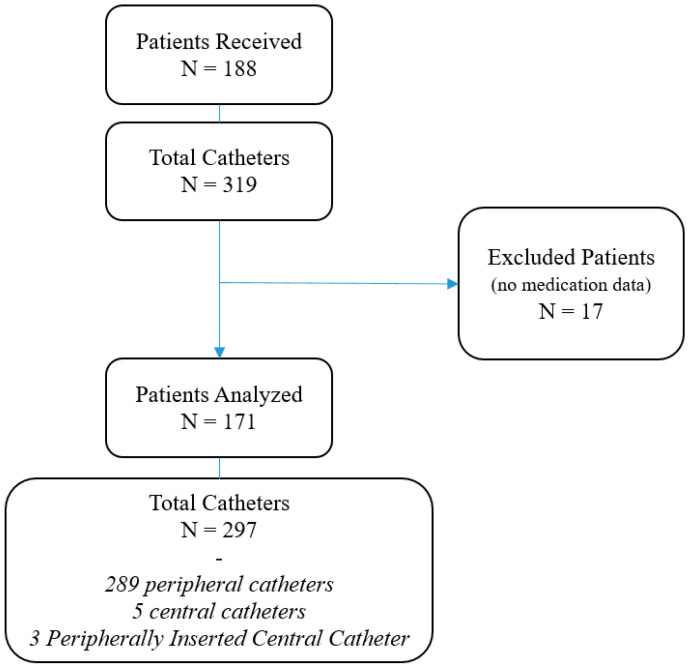
Study flowchart.

One hundred and eighty-eight patients were included, with 319 catheters inserted by 68 nurses. Seventeen patients (8.8%) were ruled out due to the lack of data on the medication administered, resulting in 171 patients being included in the final analysis, with 174 episodes and 297 catheters inserted (Figure 1). The mean patient age was 70.5 years [SD 14.6, Median 74.0 (Q1; Q3 60.0; 82.0)]; 90 were women (52.6%).

### 3.2. Catheters

The total amount of inserted catheters was 297, of which 289 (97.3%) were SPC, five (1.7%) CICC, and three (1.0%) PICC. A total of 223 catheters (75.3%) were used to administer high-risk medication; only three were adequately chosen (1.4%) CI [0.3–3.9]. Of the 289 SPC, 48 were adequate (16.6%) CI [12.5–21.4], and of eight central catheters (PICC or CICC), three (37.5%) were adequate CI [8.523–75.514]. A median of two catheters [IQR: 1–2] was used per patient and hospital admission. The data for the analysis of the number of catheters and adequacy by service, admission days, and medication-associated risk are listed in Table 1. Most frequently, a catheter was inadequate when medication was high risk and for lengthier hospitalizations. The results of the mixed logistic models are shown in Table 2. Based on our results, none of the factors studied was associated with the right or wrong choice of the catheter.

In the group of catheters selected according to published recommendations (*n* = 51), complications occurred in 32 catheters (62.7%). In the group of catheters not selected according to recommendations (*n* = 246), complications occurred in 157 catheters (63.8%). The difference between groups was not statistically significant (*p* = 1.0).

Omeprazole was prescribed to 212 patients in our cohort. It is considered a high-risk medication and is taken by most hospitalized patients to prevent stress ulcers; however, it is frequently administered through a PVC. If omeprazole is excluded from the analysis, the use of the SPC would be appropriate in 66.5% of cases (see Supplementary Materials).

### 3.3. Nurses

The number of participating nurses was 68, with a mean age of 37.9 years (SD 9.0, range of 30–44); 59 were women (86.8%). The demographic characteristics of the nurses participating in the study are detailed in Table 3. The mean proportion of adequately inserted catheters per nurse was 22% (95% CI [14–30%]). Twenty-three nurses responded to the self-administered questionnaire about their autonomy in choosing the catheter, and all 23 respondents (100%) reported being always or almost always autonomous in choosing the type of venous catheter. The Poisson Model of nurses’ adequacy is detailed in Table 4. Variables analyzed related to nursing staff showed no significant contribution to the adequacy of the venous catheter used.

## 4. Discussion

The results from this cross-sectional study, which included inpatients with venous catheters, showed that the majority of catheters inserted during the study were inadequate, based on the number of days with inserted catheters and the medication risk prescribed. Inadequacy was higher when risk medication or intravenous treatment for prolonged periods was used. More specifically, inadequacy was more frequent in catheters used to administer high-risk intravenous drugs (according to pH/osmolarity) and in those maintained for prolonged IV treatments (7 or more days), situations in which a central device or PICC would be recommended. Notably, omeprazole accounted for the majority of cases in which the catheter type was incorrectly selected. In addition, an average of two catheters per admitted patient (episode) was used when it would have been sufficient to use only one catheter per patient if the established recommendations had been followed.

Omeprazole is classified as a high-risk intravenous medication because of its alkaline pH and, according to published recommendations, it would therefore be preferentially administered through a central venous access. However, in routine clinical practice, omeprazole is prescribed to a large proportion of hospitalized patients—often as stress-ulcer prophylaxis—and central venous access is not inserted solely for the purpose of administering this drug. If guideline recommendations were applied strictly in this context, a substantial number of inpatients would require a central line, which is neither realistic nor clinically justified in most cases. This illustrates a marked and widespread divergence between guideline-based criteria and everyday practice. This discrepancy likely reflects the fact that current recommendations are primarily based on pharmacological properties of medications, without fully accounting for clinical feasibility, risk proportionality, or the widespread use of certain drugs in routine care. In practice, clinicians may reasonably prioritize avoiding unnecessary central venous access, accepting a theoretical increase in local risk in favor of reducing invasiveness and potential central-line–related complications. From this perspective, part of the inadequacy observed in our study may represent a limitation of guideline applicability in real-world settings rather than suboptimal clinical decision-making. Importantly, the frequent inadequacy in catheter selection observed in our study extends beyond omeprazole: even in the sensitivity analyses excluding omeprazole, the proportion of adequately selected catheters remained low, indicating that suboptimal catheter selection is a broader issue rather than being driven only by this medication.

Beyond catheter selection, another aspect that may contribute to the high prevalence of intravenous therapy observed in our cohort is the initial choice of the route of administration for certain medications. In routine practice, intravenous omeprazole is often initiated at hospital admission and subsequently switched to oral therapy once oral intake is tolerated. However, in some patients who are able to tolerate oral medications from the beginning of hospitalization—as evidenced by the concomitant use of other oral drugs—omeprazole could potentially be prescribed orally from admission. If this is not consistently implemented, the initial use of the intravenous route may lead to unnecessary vascular access and prolonged catheter use. Although our study was not designed to evaluate the appropriateness of the route of administration, optimizing early oral versus intravenous prescribing may represent an additional opportunity to reduce unnecessary catheter placement and improve overall catheter adequacy.

None of the factors analyzed related to the patient or the nurse were associated with the inadequacy of the catheter, not even the nurse’s experience or training in a specific program. However, the confidence interval for training was wide, likely due to the small number of trained nurses and limited power, so a true effect cannot be ruled out. Standardized training, combined with decision-support tools, may still improve adherence to catheter-selection recommendations and should be assessed in larger studies.

We have not found any study that evaluates the correct selection of the type of catheter used, depending on the characteristics of the medication and the administration time. On the other hand, other authors have assessed whether the number of vascular catheters placed in hospitalized patients was adequate. Fernandez-Ruiz et al. investigated the number of catheters and lumens available to patients [22], depending on whether all the medication could be administered through the same catheter or if there was a medication that required an exclusive line, and also whether the patient needed catheters for preventive reasons (patients at risk of instability). They found that of the 571 patients studied, 126 (21.9%) had an inadequate number of catheters [22].

Tiwari et al., in a prospective study conducted in an adult medical–surgical ward at the Nebraska Medical Center, found that of the 3806 total catheter-days recorded, 1179 (31%) were inappropriate [23]. The authors considered appropriate any catheter used at least once every 24 h to administer medication, blood, fluids or parenteral nutrition, hemodynamic monitoring, or intravenous access in cardiac arrhythmias or for dialysis. They also considered appropriate catheters that were kept for prevention if they had been used at least once in the previous 48 h. However, these authors did not analyze whether the type of catheter was appropriate for the medication or the treatment time, so our results are not comparable.

All the nurses who completed the survey on autonomy recognized that they were autonomous when deciding what type of catheter to use. However, given the inappropriateness of the figures obtained, it can be inferred that there is a lack of information or training that would allow them to make this choice adequately. In routine practice, the expected duration of intravenous therapy is often uncertain at the moment of catheter insertion and may change as diagnostic information becomes available and the patient’s response to treatment evolves. Therefore, beyond clinical judgment and periodic reassessment of the vascular access, a more feasible improvement would be to integrate into the Hospital Information System automatic alerts from the pharmacy regarding drug-related risk (e.g., extreme pH or high osmolarity) and the recommended type of venous access for each medication. In this context, closer involvement of clinical pharmacists in monitoring treatment duration and drug-related risk, particularly in vulnerable patients, could further enhance the safety and appropriateness of intravenous therapy. Importantly, such alerts should be conceived as decision-support tools rather than prescriptive rules. By providing contextualized, real-time information at the point of care, they may help clinicians reassess vascular access choices as treatment evolves, particularly when initial decisions were made under uncertainty regarding treatment duration. In this way, electronic alerts could help bridge the gap between guideline-based recommendations and real-world practice, supporting proportional and informed decision-making without undermining professional autonomy. Ensuring easy access to updated decision algorithms, together with targeted training and professional development programs, could further support nurses in selecting the most appropriate catheter.

It has been shown that the development of training programs for the correct use and care of intravenous lines has a positive impact on adverse events (i.e., phlebitis and infections), although there are still few studies showing these results. Compliance is easy to control through point prevalence studies, mainly in patients not admitted to the ICU [24] (Pérez-Granda et al., 2015).

One relevant limitation of the study was that there was no time forecast for the duration of treatment with intravenous medication, so it is possible that the catheter was adequate at the time of insertion for the standard time predicted for each type of medication, but it had to be later extended because the patient needed intravenous medication for a longer time. The physician’s initial forecast was unavailable because they did not record it in the medical record, so the nurses probably did not have this information when selecting the catheter. This limitation reflects the usual clinical practice, in which the catheters are chosen without having a forecast of the medication administration time indicated by the physician. From a quality improvement perspective, these findings could inform educational interventions targeting both prescribing physicians and nursing staff responsible for catheter insertion, fostering a shared and coordinated approach to intravenous therapy management.

In addition, the questionnaire used to assess nurses’ autonomy and knowledge was developed ad hoc and had not been formally validated. As no validated instruments addressing this specific topic were available, the results derived from this questionnaire should be interpreted with caution, as they may be subject to limitations in precision and validity.

Intensive care unit and emergency department patients were excluded from this study. These settings have distinct clinical priorities and vascular access practices, and complete comparable data were not available for analysis. Therefore, our findings are primarily applicable to acute inpatient wards and may not be generalizable to critical care or emergency settings. Nonetheless, the implementation of decision-support tools and automated alerts within the Hospital Information System could potentially support protocol adherence in time-pressured environments such as the emergency department.

Another limitation is the small sample size in some sub-analyses. In particular, central catheters were infrequent in our cohort (only eight devices), which resulted in wide confidence intervals and limited precision for this subgroup. The study was not powered to perform comparisons between different types of venous catheters, as the sample size was calculated to estimate the overall proportion of adequate catheter selection. Therefore, any comparisons involving central catheters should be interpreted cautiously, and larger studies with a higher number of central devices are needed to draw robust conclusions about adequacy by catheter type.

Finally, it is interesting to note that we were unable to prove a higher number of complications in the group of catheters that had been selected against the previously published recommendation. Although our study does not have the power to test this hypothesis, confirmation of these data in more extensive studies would point to the lack of efficacy of existing recommendations to prevent catheter-associated complications in hospitalized patients.

## 5. Conclusions

This study is one of the first to analyze the adequacy of venous catheter selection in an acute care units and shows that catheters are generally not well selected, since many peripheral catheters are placed in patients who require intravenous medication over a prolonged period or are receiving high-risk medication. These findings highlight the complexity of applying guideline-based recommendations in real-world clinical settings, particularly when treatment duration is uncertain and commonly used high-risk medications are involved. These findings also highlight the need for practical strategies to support decision-making in catheter selection, such as improving the availability of information on expected intravenous treatment duration when feasible, reinforcing access to updated guidelines, and providing ongoing education and decision-support tools for nursing staff. The integration of decision-support tools and automated alerts into hospital information systems, together with closer collaboration with pharmacy services, may further support clinicians in reassessing vascular access choices as treatment evolves. Future studies should evaluate whether these interventions improve catheter adequacy and reduce catheter-related complications. Given the widespread use of venous access devices in hospitals, improving catheter-selection processes may have important implications for patient safety and healthcare quality.

## Figures and Tables

**Table 1:** Number of catheters and adequacy by service, hospitalization days and medication risk.

	All, *n*	Adequate *n*, (%)	95% CI	*p*-Value
Service	*N* = 297	*n* = 48		
Cardiology	21	11 (52.4%)	[29.8–74.3]	<0.001
Geriatrics	16	7 (43.8%)	[19.8–70.1]	
Internal Medicine	78	23 (29.5%)	[19.7–40.9]	
Pneumology	20	3 (15.0%)	[3.2–37.9]	
Urology	20	2 (10.0%)	[1.2–31.7]	
Neurology	17	1 (5.9%)	[0.2–28.7]	
General Surgery	69	1 (5.8%)	[1.6–14.2]	
Traumatology	56	0 (0.0%)	[0.0–6.4]	
Admission days	*N* = 297	*n* = 51		
≤2 days	84	18 (21.4%)	[14.0–31.3]	<0.001
2–4 days	77	20 (26.0%)	[17.5–36.7]	
4–6 days	56	11 (19.6%)	[11.3–31.8]	
>6 days	80	2 (2.50%)	[11.3–31.8]	
Medication Risk	*N* = 296	*n* = 50		
Low risk	73 (24.7)	47 (64.4)	[52.3–75.3]	<0.0001
High risk	223 (75.3)	3 (1.4)	[0.3–3.9]	

Abbreviations: n, number; CI, confidence interval.

**Table 2:** Mixed logistic models including the variables associated with the catheter, patients, and nursing.

Mixed Logistic Models				
Predictors	OD	SE	CI	*p*
(Intercept)	0.00	0.00	0.00–0.00	<0.001
Patients				
Sex [Man]	0.9	1.8	0.0–48.5	0.942
Age	1.6	2.0	0.1–18.7	0.727
Nurses				
Sex [Man]	0.1	0.5	0.0–559.6	0.620
Age	1.4	1.2	0.3–7.6	0.718
Years of Experience	0.9	0.2	0.7–1.3	0.630
Training [Yes]	1.8	2.3	0.2–21.0	0.637
Random Effects				
σ^2^	3.3			
τ00 Patient ID	1180.4			
τ00 Service	4.4			
ICC	1.0			
N Patient ID	157			
N service	8			
Observations	264			
Marginal R^2^/Conditional R^2^	0.001/0.997			
AIC	139.0			

Abbreviations: OD, Odds ratio; SE, Standard error; CI, confidence interval; σ^2^, population variance; τ00, between-subject-variance; R^2^, r-squared; AIC, Akaike information criterion.

**Table 3:** Demographic and professional characteristics of participating nurses.

Nurses’ Characteristics		
*N* = 74	*n*, (SE)	Median, [Q1; Q3]
Sex (*n* = 68)		
Woman	59 (86.8%)	
Man	9 (13.2%)	
Age (*n* = 69)	37.9 (9.0)	37.0 [30.0; 44.0]
Years of experience (*n* = 69)	9.5 (6.8)	10.2 [3.6; 13.8]
Participation in a training program (*n* = 74)		
Yes	17 (23.0%)	
No	57 (77.0%)	

Abbreviations: SE, standard error; N, number.

**Table 4:** Poisson Model of nurses’ proportion of adequately inserted catheters.

	Nurses’ Adequacy Rate			
Predictors	Incidence Rate	SE	CI	*p*-Value
(Intercept)	0.2	0.1	0.0–0.8	0.932
Sex [man]	1.3	0.5	0.6–2.5	0.242
Age	1.0	0.0	0.9–1.0	0.347
Years of experience	1.1	0.0	1.0–1.1	0.622
Training [Yes]	0.9	0.3	0.5–1.6	0.976
Observations	68			
R^2^ Nagelkerke	0.124			
AIC	148.2			

Abbreviations: SE, standard error; CI, confidence interval; R^2^, r-squared; AIC, Akaike information criterion.

## Data Availability

The datasets used and/or analysed during the current study are available from the corresponding author on reasonable request.

## Supplementary Materials

The following supporting information can be downloaded at: https://www.mdpi.com/article/10.3390/nursrep16020076/s1, Table S1. Number of peripheral and central catheters and adequacy, excluding omeprazol. Table S2. Number of catheters and adequacy by service, excluding omeprazol.

## Public Involvement Statement

No public involvement in any aspect of this research.

## Guidelines and Standards Statement

This manuscript was prepared in accordance with the Strengthening the Reporting of Observational Studies in Epidemiology (STROBE) guidelines.
