# Supplementary material for: Adequacy of the Type of Venous Catheter to the Drug Type and Duration of Treatment: A Cross-Sectional Study
Source: Nurs Rep. 2026 Feb 21;16(2):76. doi: 10.3390/nursrep16020076 (PMC12943557; doi:10.3390/nursrep16020076)
Supplement: Supplementary file 1 [file nursrep-16-00076-s001.zip › nursrep-4074518-supplementary.pdf]

Supplementary material

Table S1. Number of peripheral and central catheters and adequacy, excluding omeprazol.

	**All, n**	**Adequate n, (%)**	**95% CI**
Peripheral	287	191(66.5%)	[60.768–71.987]
Central	8	3 (37.5%)	[8.523–75.514]

Table S2. Number of catheters and adequacy by service, excluding omeprazol.

	**All, n**	**Adequate n, (%)**	**95% CI**	***P*-value**
**Service**	**N = 295**	**n = 194**		
Traumatology	56	44 (78.57%)	[65.56-88.41]	
Cardiology	20	14 (70.00%)	[45.72–88.11]	
General Surgery	69	47 (68.12%)	[55.80-78.83]	
Internal Medicine	78	52 (66.67%)	[55.08-76.94]	0.0335
Neurology	16	10 (62.50%)	[35.44-84.80]	
Geriatrics	16	10 (62.50%)	[35.44-84.80]	
Pneumology	20	10 (50.00%)	[27.20-72.80]	
Urology	20	7 (35.00%)	[15.39-59.22]	
